# Factors associated with dietary adherence to the guidelines for prevention and treatment of hypertension among Korean adults with and without hypertension

**DOI:** 10.1186/s40885-020-00138-y

**Published:** 2020-03-15

**Authors:** Jee-Seon Shim, Ji Eun Heo, Hyeon Chang Kim

**Affiliations:** Department of Preventive Medicine, Yonsei University College of Medicine, Yonsei-ro 50-1, Seodaemun-gu, 03722 Seoul, Republic of Korea

**Keywords:** Hypertension, Diet therapy, Self-management, Knowledge, Attitudes, Practice, Therapeutic adherence and compliance, Self-efficacy

## Abstract

**Background:**

Although dietary modification is strongly recommended for prevention and treatment of hypertension, little is known about which factors are associated with adherence to dietary guidelines. We investigated knowledge and attitude, perceived benefits of, barriers to, and self-efficacy of dietary therapy, and identified the factors associated with dietary adherence among adults with and without hypertension.

**Methods:**

We collected information on the knowledge/attitudes and perceived benefits of dietary therapy, as well as barriers to and self-efficacy regarding dietary adherence from 497 middle-aged (34–69 years) adults who participated in the follow-up examination of the Cardiovascular and Metabolic Diseases Etiology Center (CMERC) cohort study during December 2018 and February 2019.

**Results:**

Among people without hypertension, 95.5% (343/359) and 95.8% (344/359) answered that they would limit sodium intake and consume health diet, respectively, if diagnosed hypertension. However, among people with hypertension, only 79.7% (110/138) and 77.5% (107/138) reported they were limiting dietary sodium intake and having healthy diet, respectively. Frequency of diet management was not different between normotensive (34.0%) and hypertensive (35.5%) groups. Compared to normotensives, hypertensive people were more likely to have lower dietary adherence score, think they need to change their diet, think dietary change impossible, and report lower self-efficacy for following diet guidelines. Dietary management was significantly associated with cardiometabolic risk factors (OR: 1.63) and dietary education (OR: 2.19) among normotensives, while it was associated only with awareness that lifestyle modification is necessary regardless of antihypertensive medication (OR: 6.29) among hypertensive people. Good dietary adherence had significant associations with perceived barriers (OR: 0.71), self-efficacy (OR: 3.71), and dietary education (OR: 1.98) among normotensives; and with perceived barriers (OR: 0.54), self-efficacy (OR: 4.06), and dietary management (OR: 4.16) among hypertensive people.

**Conclusions:**

Many Koreans have relatively low adherence to dietary guidelines for hypertension prevention and treatment. Knowledge, dietary practices, and factors affecting dietary adherence were different between adults with and without hypertension. A targeted approach will be needed to improve blood pressure control of the Korean population.

## Background

Hypertension is a major health burden worldwide [1]. The lifetime risk for hypertension for middle-aged and older individuals has been estimated to be as high as 90% and the prevalence of hypertension has steadily increased with ageing of populations [1, 2]. Uncontrolled blood pressure (BP) has been a major contributor to cardiovascular diseases, stroke, end-stage renal diseases, and the leading cause of death [1, 3, 4]. However, elevated BP is effectively modifiable through antihypertensive medication and lifestyle modifications such as dietary sodium restriction, alcohol moderation, and regular exercise [5, 6]. Healthy lifestyle can reduce BP without any harmful side effects, and in addition, can improve overall cardiovascular health [7, 8]. These benefits of lowered BP have been identified in individuals who have not yet entered the stage where clinical care is required but are still at risk for developing hypertension, as well as those previously diagnosed with hypertension [6, 9, 10]. Thus, lifestyle modification is strongly recommended for hypertensive patients regardless if they are being treated with antihypertensive medications, as well as for normotensive individuals [5, 6].

However, guidelines for lifestyle modification are not widely followed [11–13]. Despite the well-known benefits of dietary intervention, compliance with dietary guidelines for hypertension is poorest for health-related behaviors such as non-smoking, alcohol consumption in moderation, and physical activity [13, 14], and unhealthy diet has been reported to be a major barrier to disease management [15]. Several studies have been conducted to gain insight into the knowledge and disease management practices of individuals with hypertension and/or diabetes. However, individuals face unique difficulties depending on their culture, health-care system, food prices, and economic status in the society they live in [15, 16]. Little is known about which difficulties are prevalent among adults in the South Korean population and whether or not there are gaps in knowledge and dietary practices of individuals with and without hypertension. Thus, in this study, we evaluated knowledge and attitude, perceived benefits of, barriers to, and self-efficacy of dietary therapy, and identified the factors associated with dietary adherence among adults with and without hypertension.

## Methods

### Data source and study population

This study was based on the Cardiovascular and Metabolic Diseases Etiology Research Center (CMERC) cohort study. The design, methods, and rationale of the CMERC cohort study have been reported elsewhere [17]. Briefly, the CMERC cohort was initially designed as a community-based general population cohort study to identify new risk factors and to develop evidence-based prevention strategies for cardiovascular and metabolic diseases in Korea. The cohort comprises community-dwelling middle-aged (age 30–64) adults who were free of cardiovascular diseases. Recruitment and baseline examination had been carried out between 2013 and 2018 at two research clinics, Yonsei University College Medicine in Seoul (*n* = 4060) and Ajou University School of Medicine (*n* = 4037) in Suwon, Korea.

The present analysis used data from a follow-up survey at Yonsei University research clinic between December 2018 and February 2019, since dietary adherence questionnaire was first employed at this survey. Target population for this follow-up survey was 807 adults who were recruited during the first year of enrollment period. During the follow-up period, 2 people died and 18 people withdrew their consent on study participation. Among the remaining 787 participants, a total of 500 participated in the follow-up survey, but three were excluded because of incomplete data. Eventually, 497 adults aged 34 to 69 years were analyzed in this study (Supplementary Fig. S1).

### Knowledge of and attitude towards hypertension treatment

We developed a questionnaire to assess individuals’ knowledge and attitudes toward guidelines for hypertension treatment. Questions sought to determine: (1) whether the participant was aware of the health impact of uncontrolled BP and (2) whether they were aware that lifestyle modifications are recommended even in if taking BP-lowering medications. Attitudes regarding self-management guidelines for hypertension including medication, non-smoking, abstinence from alcohol drinking, maintaining optimal body weight, exercise, limiting dietary sodium intake, and consuming a healthy diet, were also investigated [5]. Adults with hypertension were asked to evaluate whether or not they were currently following these guidelines in their daily life. Those without hypertension were asked to answer whether or not they would follow the guidelines if they were to be diagnosed with hypertension.

### Dietary self-management, benefits, barriers, and self-efficacy of dietary therapy

The presence of dietary management was assessed using the question “*Are you managing your diet for any special reason?*” that is used in the Korea National Health and Nutrition Examination Survey [18]. We surveyed how well participants had followed the individual dietary guidelines for hypertension over the previous month, i.e. reduction in dietary sodium intake, consumption of appropriate amounts of food (not overconsumption), sufficient intake of fruit and vegetables, and a well-balanced diet. Each question about dietary practice was referred to the guidelines recommended by the Korean Society of Hypertension (KSH) [5]. Dietary adherence to each guideline was scored based on a scale of 1 (strongly disagree) to 4 (strongly agree). We defined ‘*good adherence*’ as a mean score of 3 (75th percentile).

Perceived benefits of and barriers to dietary modification for hypertension were investigated. Participants were asked whether or not they experienced difficulties in preparing meals adherent to the guidelines, the taste of the food, dietary management skills, and changing their dietary habits. Self-efficacy with regard to reducing dietary sodium intake, consuming an appropriate amount of food, having a sufficient intake of fruit and vegetables, and eating a well-balanced diet was assessed by scoring these items from 1 (strongly disagree) to 4 (strongly agree). All information collected was self-reported and our questionnaires relating to diet are presented in Supplementary Fig. S2.

### Other variables

A wide range of information on health status as well as potential factors related to health was collected in the follow-up survey. All participants fasted more than 8 h prior to examination. Health examination and interviews were conducted by trained research staff according to a predefined study protocol [17].

Socioeconomic status, including education and subjective economic status, medical history including past history of disease, family history of hypertension, frequency of BP measurements over the previous year, awareness of BP values, information on hypertension (only for adults with hypertension) such as age at first diagnosis and current use of medications, and health-related behaviors including smoking, drinking, and physical activity were collected by interview.

BP of both arms was measured using an automated oscillometric device (WatchBP office, Microlife, Widnau, Switzerland). Systolic BP (SBP) and diastolic BP (DBP) were repeatedly measured at 1-min intervals after at least 5-min rest in a seated position, and the average of the three measurements was used. More details are provided elsewhere [19]. Hypertension was defined as SBP ≥ 140 mmHg, DBP ≥ 90 mmHg, or currently being treated with BP-lowering medications [5].

Weight and height were measured using a stadiometer and a digital scale, respectively, and body mass index (BMI) was calculated. Obesity was defined as BMI ≥25.0 kg/m^2^. Diabetes mellitus was defined as either a fasting glucose level ≥ 126 mg/dL or when participants reported anti-diabetic treatment, such as taking a hypoglycemic agent or insulin. Hypercholesterolemia was defined as serum cholesterol level ≥ 240 mg/dL or lipid-lowering drug use, hypertriglyceridemia as serum triacylglycerol level ≥ 200 mg/dL or lipid-lowering drug use, and low HDL-cholesterol as HDL cholesterol level < 40 mg/dL in men and < 50 mg/dL in women. If at least one among hypercholesterolemia, hypertriglyceridemia, or low-HDL cholesterol was present, we considered the participant to have dyslipidemia.

### Ethical aspects

This study was approved by the Institutional Review Board (IRB) of Severance Hospital, Yonsei University Health System, Seoul, Korea (4–2013-0661). Written informed consent was obtained from all participants prior to the survey. Study procedures were performed in accordance with the ethical standards of the responsible institutional committee on human experimentation and were in accordance with the Helsinki Declaration (of 1975 as revised in 2008).

### Statistical analyses

Participant characteristics are presented as means ± SDs or as frequencies (%). The significance of differences in means and distributions between those with hypertension and those without were assessed using t-tests and chi-square tests. The associations between study variables and self-reported dietary management behavior or dietary adherence to guidelines were analyzed by logistic regression analyses. Sex- and age-adjusted odds ratios (ORs) and multivariable-adjusted ORs and 95% confidence intervals (95% CIs) were calculated. In a multivariate model to explore the factors associated with dietary management behavior, we included sex, age (years), education (≥college or < college), cardiometabolic risk (at least one of obesity, diabetes mellitus, or dyslipidemia, or none), family history of hypertension (yes or no), awareness of BP value (aware or not aware), healthy habits (currently a non-smoker, non-drinker, and regular walking, or not), knowledge of the necessity for lifestyle modification regardless of BP-lowering drug treatment (knowledge present or absent), perceived barriers to dietary therapy (number of barriers), self-efficacy regarding following guidelines (mean score ranging from 1 to 4), dietary education over the preceding year, and perceived necessity for diet change (yes or no). To explore the factors associated with dietary adherence to hypertension guidelines, the following variables were included in the model: sex, age (years), education (≥college or < college), cardiometabolic risk (at least one of obesity, diabetes mellitus, and dyslipidemia, or none), family history of hypertension (yes or no), awareness of BP value (aware or no), healthy habits (currently a non-smoker, non-drinker, and regular walking, or not), knowledge of the necessity for lifestyle modification regardless of BP-lowering drug treatment (knowledge present or absent), perceived barriers to dietary therapy (number of barriers), self-efficacy regarding following guidelines (mean score ranging from 1 to 4), dietary education over the preceding year, and self-reported dietary management behavior (yes or no). We performed logistic regression analyses stratified by adults with and without hypertension. All analyses were performed using the statistical software package SAS (version 9.4; SAS institute, Cary, NC, USA). *P*-values < 0.05 were considered statistically significant.

## Results

### Demographic and disease-related characteristics of participants

Among 497 participants, 27.8% had hypertension (*n* = 138). Among the hypertensive adults, mean hypertension duration was 8.4 ± 6.6 years and more than 90% of individuals reported that they were taking BP-lowering drugs (Table 1). Hypertensive adults were significantly older, less educated, and had more cardiometabolic risk factors such as obesity, diabetes mellitus, and dyslipidemia than normotensive adults. In addition, more of these participants had a family history of hypertension than non-hypertensive adults. Fortunately, the hypertensive adults measured their BP more frequently than normotensive adults (*P*-value < 0.01) and their awareness of their BP value also was higher (*P*-value < 0.01). However, there was no difference in lifestyle behaviors at the time of the survey between those with and without hypertension. Experience of dietary education over the preceding year was lower in hypertensive adults than normotensive adults with borderline significance (*P*-value = 0.07).

**Table 1:** Demographic and disease-related characteristics of Korean adults with and without hypertension

	Total (*n* = 497)	Normotensives (*n* = 359)	Hypertensives (*n* = 138)	*P*-value
Sex (women)	346 (69.6)	254 (70.8)	92 (66.7)	0.44
Age (y)	56.9 ± 8.5	55.5 ± 8.8	60.7 ± 6.2	< 0.01
Education (college or higher)	195 (39.3)	155 (43.3)	40 (29.0)	< 0.01
Subjective economic status (low)	81 (16.3)	52 (14.5)	29 (21.0)	0.10
Cardiometabolic disease status				
Obesity^a^	155 (31.2)	88 (24.5)	67 (48.6)	< 0.01
Diabetes mellitus^b^	46 (9.3)	23 (6.4)	23 (16.7)	< 0.01
Dyslipidemia^c^	237 (47.7)	143 (39.8)	94 (68.1)	< 0.01
Family history of hypertension	229 (46.1)	150 (41.8)	79 (57.3)	< 0.01
Hypertension duration (years)	–	–	8.4 ± 6.6	–
Antihypertensive drug treatment (yes)	–	–	105 (93.8)	–
Frequency of BP measurement during the previous 1 year				
≤ 4 times	267 (53.7)	236 (65.7)	31 (22.5)	< 0.01
5–12 times	161 (32.4)	87 (24.2)	74 (53.6)	
> 12 times	69 (13.9)	36 (10.0)	33 (23.9)	
Awareness of their own BP value (yes)	359 (72.2)	243 (67.7)	116 (84.1)	< 0.01
Lifestyle behaviors	113 (22.7)	86 (24.0)	27 (19.6)	0.35
Current non-smoking	454 (91.4)	328 (91.4)	126 (91.3)	1.00
Current non-drinking	191 (38.4)	142 (39.6)	49 (35.5)	0.47
Regular walking (≥60 min/day)	328 (66.0)	241 (67.1)	87 (63.0)	0.45
Dietary education over the past 1 year (yes)	72 (14.5)	58 (16.4)	13 (9.4)	0.07

Abbreviations: *BP* blood pressure

Values are presented as N (%) or mean ± SD

^a^Obesity was defined as a body mass index ≥25.0 kg/m^2^

^b^Diabetes was defined as either a fasting glucose level ≥ 126 mg/dL or when participants self-reported anti-diabetic treatment, such as taking a hypoglycemic agent or insulin

^c^Dyslipidemia was defined if any one of hypercholesterolemia (serum cholesterol level ≥ 240 mg/dL or self-reported lipid-lowering drug use), hypertriglyceridemia (serum triacylglycerol level ≥ 200 mg/dL or self-reported lipid-lowering drug use), or low HDL-cholesterol (HDL cholesterol level < 40 mg/dL in men and < 50 mg/dL in women, respectively) is present

### Knowledge of and attitude towards hypertension treatment

**Fig. 1:**
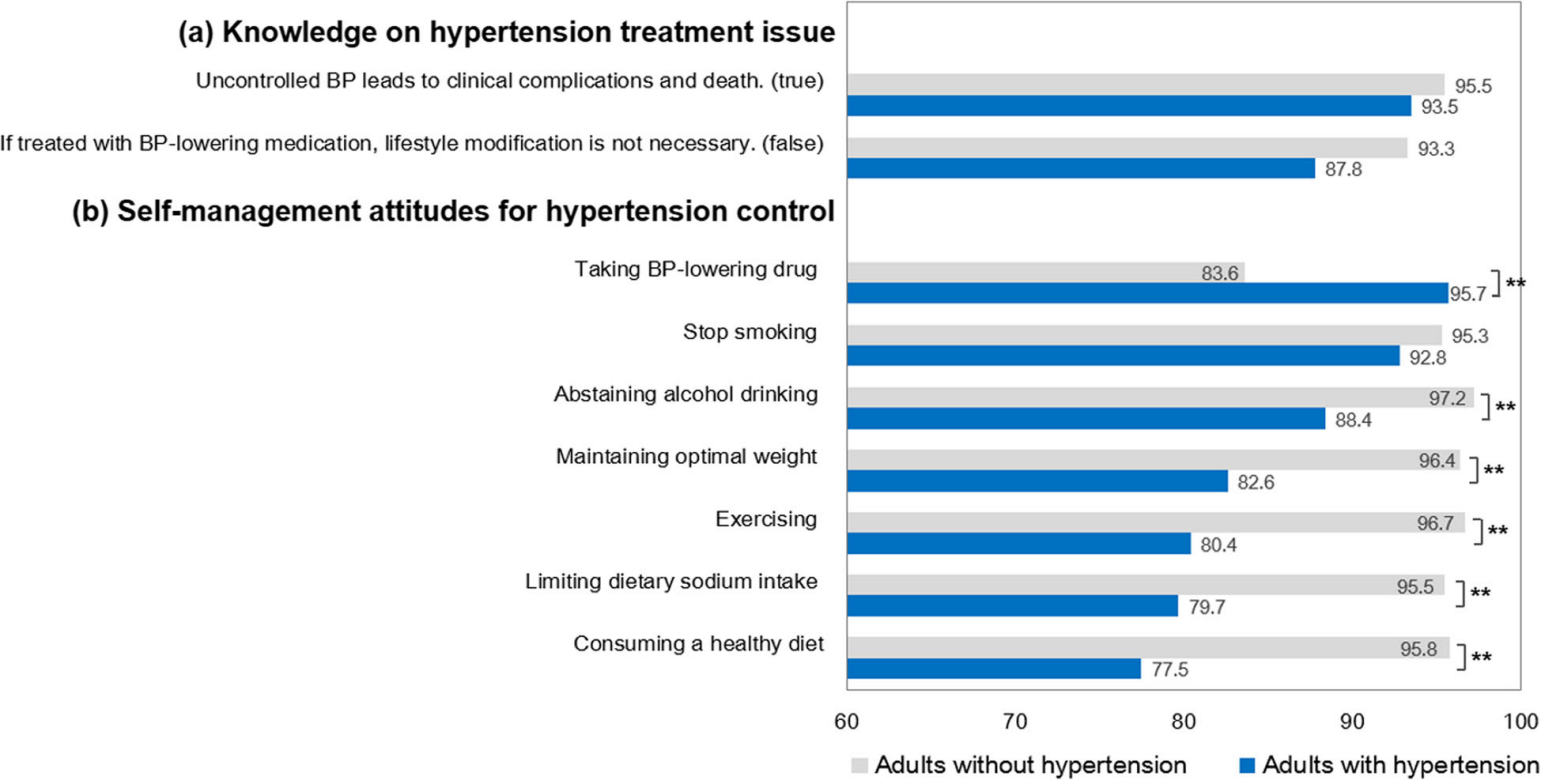
Knowledge and attitude on self-management behaviors for hypertension control. ** *P* -value < 0.01

Figure 1 shows the participants’ knowledge regarding hypertension treatment and their attitudes towards hypertension control. Almost all adults knew the harmful impact of uncontrolled BP on health, and this did not differ between those with (93.5%) and without hypertension (95.5%). Most adults also knew that lifestyle modification was necessary regardless of BP-lowering drug treatment, but the proportion of individuals with correct knowledge was slightly different between the two groups. More adults with hypertension (12.2%) answered that lifestyle modification was not necessary if treated with BP-lowering drug than those without (6.7%), although this difference did not reach statistical significance (*P*-value = 0.06).

The attitude of hypertensive adults was in striking contrast to that of normotensive adults. Nearly all normotensive adults (95.3–97.2%) responded that they would make an effort to modify their lifestyle (including smoking, drinking, physical activity, diet) and maintain an optimal body weight if they were to be diagnosed with hypertension in the future. Only 83.6% reported that they would take BP-lowering drugs. However, almost all adults with hypertension (95.7%) were on a BP-lowering medication, and only 77.5% reported that they consumed a healthy diet while 92.8% said that they had stopped smoking. Diet-related self-management was lower in this group than the normotensive group.

### Self-reported dietary management practices and dietary adherence over the past month

Nearly one in three adults reported that they managed their diet, and the prevalence of self-reported diet management was not different between adults with and without hypertension (Table 2). On the whole, scores for the dietary practice guidelines were not different between the hypertensive and normotensive groups, but hypertensive adults had a significantly lower score for sufficient consumption of fruit and vegetables than normotensive adults (*P*-value = 0.01). In addition, the mean of the dietary practice scores for the four guidelines was significantly lower in hypertensive adults than normotensive adults (*P*-value = 0.04). The prevalence of good adherence (mean dietary practice score of 3 or higher), although slightly lower in hypertensive adults, was not statistically significant between the two groups (*P*-value = 0.09).

**Table 2:** Dietary practices over the past month for blood pressure control

	Total (*n* = 497)	Normotensives(*n* = 359)	Hypertensives (*n* = 138)	*P*-value
Self-reported diet management (yes)	171 (34.4)	122 (34.0)	49 (35.5)	0.83
Dietary practice over the past month (score, range: 1–4)	2.64 ± 0.48^a^	2.67 ± 0.46	2.56 ± 0.54	0.04
I limited my daily sodium diet.	2.61 ± 0.74	2.62 ± 0.73	2.58 ± 0.78	0.60
I ate proper amount without overeating.	2.76 ± 0.65	2.80 ± 0.61	2.68 ± 0.74	0.11
I had a diet rich in fruit and vegetable.	2.68 ± 0.67	2.72 ± 0.66	2.55 ± 0.69	0.01
I had a well-balanced diet.	2.51 ± 0.67	2.53 ± 0.64	2.44 ± 0.75	0.20
Good adherence (mean dietary practice score of ≥3)	167 (33.6)	129 (35.9)	38 (27.5)	0.09

Values are presented as N (%) or mean ± SD

^a^mean of scores of 4 items (sodium reduction, proper amount of consumption, sufficient consumption of fruit and vegetable, well-balanced meal)

### Perceived benefits of and barriers to following dietary guidelines and self-efficacy

**Fig. 2:**
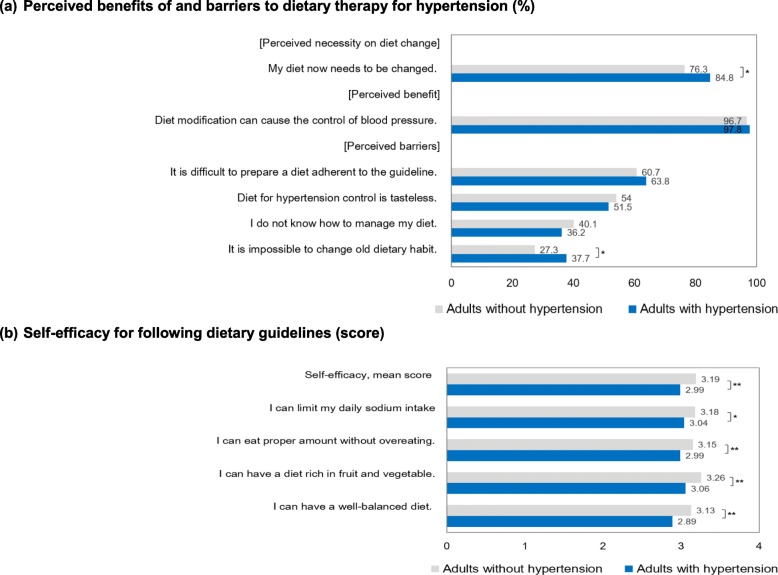
Perceived benefits of and barriers to dietary therapy for hypertension and self-efficacy for following dietary guidelines. * *P* -value < 0.05, ** *P* -value < 0.01

The majority of adults (78.8%) stated that they needed to change their current diet, and this proportion was significantly higher in hypertensive adults (84.8%, *P*-value = 0.03) (Fig. 2). Most adults, regardless if they were hypertensive or not, understood the potential benefits of dietary modification on BP control. More than half the participants felt that it was difficult to prepare a meal adherent to the guidelines (61.6%) and that BP control diets are tasteless (53.5%). Thirty-nine percent of participants did not know how to modify their diet and 30.2% of participants thought that it would be impossible for them to make dietary changes. Difficulty changing old habits was significantly more prevalent in hypertensive adults (37.7%) than normotensive adults (27.3%). Compared with adults without hypertension, those with hypertension had significantly lower self-efficacy scores for the individual guidelines and overall.

### Factors associated with dietary management and good adherence

Potential factors associated with self-reported dietary management are reported in Table 3. For adults without hypertension, dietary management behavior was significantly associated with cardiometabolic risk (OR: 1.63, *P*-value = 0.04) and dietary education (OR: 2.19, *P*-value = 0.01). For hypertensive adults, those who knew that lifestyle modification was necessary even if treated with BP-lowering drugs were 6.29-fold more likely to manage their diet (*P*-value = 0.03). Hypertensive individuals who had experienced dietary education over the preceding year tended to manage their diet more than normotensive individuals but this did not reach statistical significance (OR: 2.95, *P*-value = 0.10).

**Table 3:** Odds ratios (95% CI) for self-reported diet management behavior

		Normotensives (*n* = 359)		Hypertensives (*n* = 138)	
		Age- sex adjusted	Fully adjusted	Age- sex adjusted	Fully adjusted
Sex	(women vs. men)	1.12 (0.69, 1.83)	0.87 (0.50, 1.50)	1.63 (0.76, 3.51)	1.58 (0.66, 3.81)
Age	(y)	1.02 (1.00, 1.05)	1.02 (0.99, 1.06)	0.99 (0.93, 1.04)	0.98 (0.92, 1.05)
Education	(≥college vs. <college)	1.31 (0.80, 2.14)	1.02 (0.60, 1.72)	0.68 (0.28, 1.04)	0.61 (0.23, 1.65)
Cardiometabolic risk	(yes vs. no)	1.44 (0.92, 2.25)	1.63 (1.01, 2.63)*	1.87 (0.69, 5.09)	1.55 (0.51, 4.67)
Family history of hypertension	(yes vs. no)	1.26 (0.79, 1.98)	1.12 (0.69, 1.82)	0.77 (0.37, 1.60)	0.87 (0.40, 1.91)
Awareness of BP value	(know vs. do not)	0.84 (0.52, 1.37)	0.93 (0.56, 1.56)	0.79 (0.30, 2.10)	0.80 (0.28, 2.27)
Healthy habit	(yes vs. no)	1.27 (0.76, 2.14)	1.32 (0.77, 2.27)	0.89 (0.35, 2.23)	1.11 (0.41, 2.99)
Knowledge on the necessity for lifestyle modification regardless of BP-lowering drug use	(correct vs. wrong)	2.93 (0.97, 8.84)	2.60 (0.80, 8.42)	4.71 (1.02, 21.7)*	6.29 (1.23, 32.3)*
Perceived barriers to dietary therapy	(the number of barriers)	0.85 (0.71, 1.01)	0.84 (0.68, 1.04)	1.08 (0.82, 1.42)	1.09 (0.78, 1.53)
Self-efficacy	(score)	1.56 (0.99, 2.50)*	1.51 (0.93, 2.46)	1.38 (0.68, 2.81)	1.35 (0.60, 3.01)
Dietary education	(yes vs. no)	2.02 (1.14, 3.58)*	2.19 (1.20, 4.02)*	2.31 (0.72, 7.38)	2.95 (0.80, 10.9)
Perceived necessity for diet change	(yes vs. no)	1.46 (0.85, 2.50)	1.61 (0.90, 2.89)	1.81 (0.61, 5.35)	1.99 (0.61, 6.50)

*p value < 0.05, ** < 0.01

Cardiometabolic risk was defined if there was at least one of obesity, diabetes mellitus, or dyslipidemia

Healthy habit was defined if it was satisfied with all of current non-smoking, non-drinking, and regular walking

Factors associated with dietary good adherence to hypertension guidelines are presented in Table 4. For hypertensive adults, higher self-efficacy (OR: 4.06, *P*-value = 0.02) and the presence of dietary management (OR: 4.16, *P*-value = 0.004) and lower perceived barriers to dietary therapy (OR: 0.54, *P*-value = 0.004) were significantly associated with good dietary adherence. Similar associations were found in normotensive adults: self-efficacy (OR: 3.71, *P*-value< 0.001) and dietary education (OR: 1.98, *P*-value = 0.04) had a positive association with good dietary adherence while perceived barriers to dietary therapy (OR: 0.71, *P*-value = 0.002) was negatively associated with good dietary adherence.

**Table 4:** Odds ratios (95% CI) for good adherence to the guidelines

		Normotensives (*n* = 359)		Hypertensives (*n* = 138)	
		Age- sex adjusted	Fully adjusted	Age- sex adjusted	Fully adjusted
Sex	(women vs. men)	1.06 (0.66, 1.72)	0.81 (0.46, 1.43)	0.70 (0.32, 1.52)	0.41 (0.14, 1.21)
Age	(y)	1.02 (0.99, 1.05)	1.03 (1.00, 1.07)	1.03 (0.96, 1.10)	1.05 (0.95, 1.15)
Education	(≥college vs. <college)	1.51 (0.93, 2.46)*	1.17 (0.68, 2.03)	1.98 (0.81, 4.83)	1.34 (0.45, 3.95)
Cardiometabolic risk	(yes vs. no)	0.63 (0.40, 0.97)*	0.74 (0.45, 1.22)	0.65 (0.26, 1.64)	1.05 (0.31, 3.51)
Family history of hypertension	(yes vs. no)	1.32 (0.84, 2.07)	1.19 (0.71, 1.97)	0.99 (0.46, 2.16)	1.29 (0.50, 3.34)
Awareness of BP value	(know vs. do not)	0.64 (0.40, 1.05)	0.72 (0.42, 1.24)	1.02 (0.37, 2.87)	1.74 (0.51, 5.94)
Healthy habit	(yes vs. no)	1.15 (0.69, 1.93)	1.17 (0.66, 2.07)	1.70 (0.67, 4.30)	1.61 (0.52, 5.04)
Knowledge on the necessity for lifestyle modification even when BP-lowering drug use	(correct vs. wrong)	1.85 (0.71, 4.83)	0.93 (0.29, 3.00)	0.96 (0.31, 2.95)	0.27 (0.06, 1.14)
Perceived barriers to dietary therapy	(the number of barriers)	0.62 (0.51, 0.75)**	0.71 (0.57, 0.88)**	0.50 (0.35, 0.71)**	0.54 (0.36, 0.82)**
Self-efficacy	(score)	4.25 (2.53, 7.13)**	3.71 (2.11, 6.51)**	4.73 (1.87, 12.0)**	4.06 (1.28, 12.9)*
Dietary education	(yes vs. no)	2.00 (1.13, 3.55)*	1.98 (1.03, 3.80)*	1.19 (0.34, 4.17)	1.05 (0.25, 4.48)
Self-reported diet management	(yes vs. no)	1.96 (1.25, 3.08)**	1.61 (0.97, 2.68)	2.57 (1.17, 5.65)*	4.16 (1.58, 11.0)**

*p value < 0.05, ** < 0.01

Good adherence to the guidelines was defined as ≥3 score of the mean adherence score to 4 guidelines including reduction in dietary sodium intake, consumption in appropriate amount (not overconsuming), sufficient intake of fruit and vegetables, and a well-balanced diet

High dietary Cardiometabolic risk was defined if there was at least one of obesity, diabetes mellitus, or dyslipidemia

Healthy habit was defined if it was satisfied with all of current non-smoking, non-drinking, and regular walking

## Discussion

In this study, we investigated adherence to dietary recommendations for hypertension and identified the factors associated with dietary adherence among community-dwelling adults with and without hypertension. We found that approximately 30% individuals managed their diet, but the prevalence of diet management was not different between adults with and without hypertension, and dietary adherence score was much lower in those with hypertension than in those without. Such a low dietary adherence has been reported in previous studies; hypertensive patients showed the best adherence to medication, followed by lifestyle modifications such as drinking and physical activity, and was the poorest for diet [14, 20–22]. In particular, hypertensive patients taking BP-lowering drugs had much unhealthier lifestyles than those who were not taking medications [14, 23].

People with hypertension know that they are supposed to reduce their sodium intake, but often do not follow this dietary guideline [24]. The majority of our participants knew the necessity of lifestyle modification (91.8%) and understood that diet modification would improve BP control (97.0%), but the proportions of those who thought that their current diet needed to be changed (78.7%) and those who responded managing their diets (34.4%) were not consistent with the level of knowledge regarding hypertension treatment. In general, the consequence of not taking medication is relatively instant and distinct, whereas the impact of not modifying lifestyle appears to be less immediate and much smaller. For this reason, some people may have no expectation for nonpharmacological treatment [14] or may not feel the need for adopting a healthy lifestyle [20]. There also seems to be the incorrect belief that that a powerful drug can sufficiently address the harmful effects of unhealthy habits due to a lack of understanding regarding the mechanisms of BP regulation [25]. In our study, not a few participants thought that lifestyle modification is not required if taking medication and this trend was found more in adults with hypertension.

We also found that fewer perceived barriers were significantly associated with good dietary adherence, in both hypertensive and normotensive adults. Qualitative studies have been conducted to understand barriers to changing diet to manage hypertension [15, 16, 23]; one obstacle to dietary compliance is that people have difficulty in changing dietary habits formed over a lifetime [15]. The cost of healthy food, limited availability of appropriate foods, problems in choosing more appropriate foods when eating out, and the cumbersomeness of preparing a low salt diet when cooking meals for family members without hypertension have also been reported to be barriers to dietary change [15, 16, 24]. In our study, one of every three participants thought that it was impossible to change old dietary habits. A considerable number of participants said difficulty in preparing a diet adherent to the hypertension guidelines or did not know how to modify their diet, and more than half mentioned that a hypertension diet was not palatable. Korean sodium consumption has decreased slightly, but is still almost twice the amount recommended by the KSH [26], foods high in sodium, such as instant noodles, Kimchi, soups, and stews are frequently consumed by Koreans [27], and thus adopting a low sodium diet may be a big challenge. However, taste preference is formed as a result of adaptation to what is familiar [28]. Reducing dietary salt intake is initially difficult for most, but once habituated to low salt foods, salty taste preference can be shifted [29]. Effective strategies and consistent efforts to correct misunderstandings as well as lower the perceived barriers to dietary modification and to improve confidence are needed.

To successfully adopt healthy dietary habits, high level of self-efficacy is required. Self-efficacy, the belief in one’s ability to organize and take specific actions, has been known as a strong predictor of the action required to produce desired outcomes [30]. In our study, higher self-efficacy was positively associated with dietary adherence in both adults with and without hypertension.

Interestingly, our study showed a distinct gap between the attitude of normotensive adults towards hypertension treatment and the hypertensive adults’ actual management practices. More than 90% of adults without hypertension said they would change their lifestyle if they were diagnosed with hypertension in the future. However, among those diagnosed with hypertension, the practice rate of lifestyle modification was much lower. Previous studies have focused on patients with hypertension and/or cardiometabolic diseases [11, 14, 16, 20], thus little is known about the differences between healthy and diseased individuals. It is difficult to explain directly why fewer hypertensive adults followed lifestyle modification guidelines than normotensive individuals. Perhaps hypertensive adults tried to change their unhealthy lifestyles but failed to. The strong motivation of normotensive adults to modify their lifestyle and the relatively low level of self-management practices of hypertensive patients should be considered when physicians and healthcare providers encourage BP control through lifestyle modification.

In addition to aforementioned individuals-related factors (such as knowledge, attitude, and barriers), physician, health care system and society related factors have been identified as obstacles towards following dietary guidelines in daily life. A previous study [31] reported that physicians’ perception of hypertension treatment did not correspond to their practice. In the primary care setting, most physicians assessed patients’ medication adherence, but provided limited counselling on lifestyle modification [32]. Time constraints and inappropriate compensation were the major obstacles affecting optimal BP control through diet change, and insufficient use of diet services, even in cases where referral to a dietitian was possible, were identified as physician-related barriers [24, 32, 33]. Thus, initiatives to mitigate obstacles to preventive diet counseling and education are needed to reduce the burden caused by elevated BP. Patients with diseases that require self-management skills need to be prioritized and continuity of care needs to be improved. In addition, the social environment may assist patients, and future public campaigns are recommended [15, 16].

Our study has several limitations and strengths. First, our subjects do not represent Korean adults. Thus, cares should be taken not to make a generalization error when interpreting the results. Second, the sample size was relatively small. We were unable to analyze adults previously diagnosed with hypertension and those with newly developed hypertension separately. However, our results remained unchanged when we limited the analysis to adults previously diagnosed with hypertension and normotensive individuals. Third, we assessed the presence of dietary management behaviors and of following the dietary guidelines in a self-report manner. In general, information collected by self-report has been known to be less accurate than objective assessment. Although it is well known that self-reported adherence to lifestyle behaviors can be overestimated [34], the low proportion of individuals who managed their diets in this study indicates the need for more aggressive countermeasures. A number of previous studies that have investigated barriers to compliance have been conducted by qualitative interviews such as focus groups [15, 16, 24], or have focused on a small numbers of individuals affected with hypertension and/or cardiometabolic diseases [14–16, 20]. No information regarding the difficulties encountered by adults or the gaps between individuals with and without hypertension was provided in these previous studies. We observed a distinct difference between normotensive peoples’ attitudes towards hypertension treatment and hypertensive adults’ current behaviors regarding hypertension treatment.

## Conclusions

Many Korean adults have low adherence to dietary guidelines for the prevention and treatment of hypertension. We found that knowledge, practice, and factors associated with dietary adherence were different between those with and without hypertension. To establish more effective strategies to control blood pressure at a population-level scale, it is important to understand these gaps.

## Supplementary information

**Additional file 1: Figure S1.** Flow in the study subjects. **Figure S2.** Questionnaire on dietary adherence, management, benefits, barriers, self-efficacy of dietary therapy
