# Supplementary material for: Factors associated with dietary adherence to the guidelines for prevention and treatment of hypertension among Korean adults with and without hypertension
Source: Clin Hypertens. 2020 Mar 15;26:5. doi: 10.1186/s40885-020-00138-y (PMC7073010; doi:10.1186/s40885-020-00138-y)
Supplement: Supplementary file 1 — Additional file 1: Figure S1. Flow in the study subjects. Figure S2. Questionnaire on dietary adherence, management, benefits, barriers, self-efficacy of dietary therapy [file 40885_2020_138_MOESM1_ESM.docx]

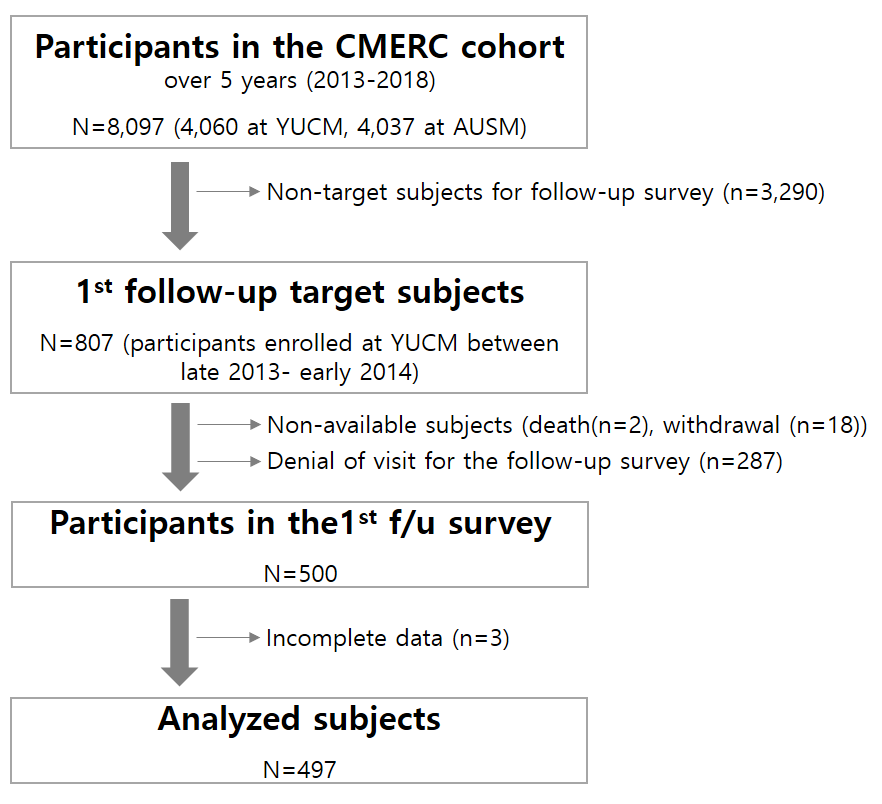


Supplementary Figure 1. Flow in the study subjects

| 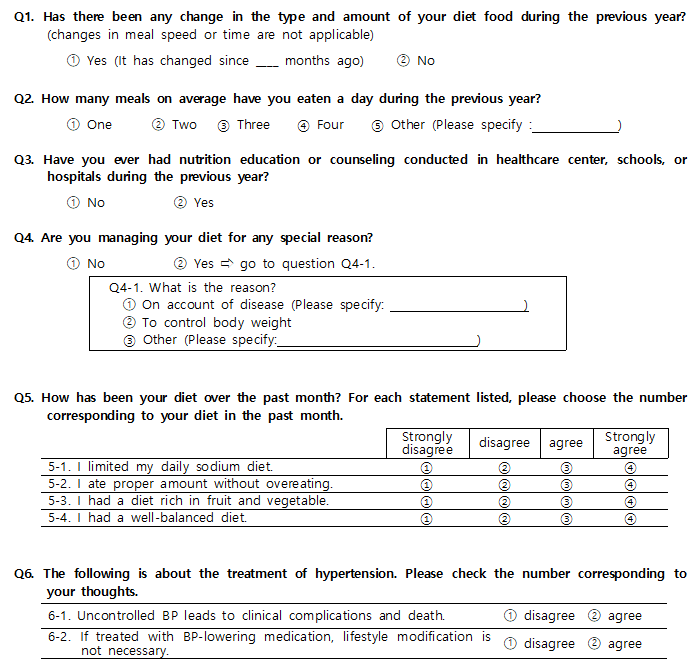 | 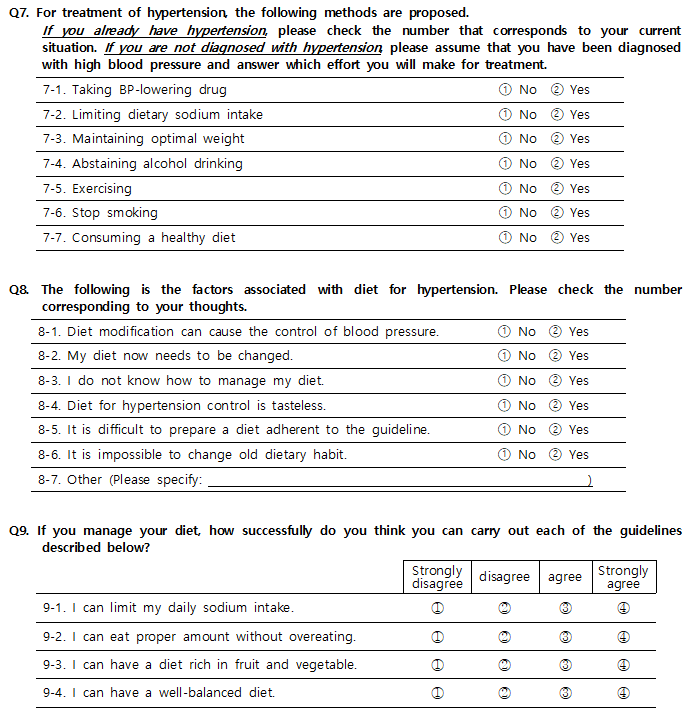 |
| --- | --- |

Supplementary Figure 2. Questionnaire on dietary adherence, management, benefits, barriers, self-efficacy of dietary therapy
